# Gene function and expression level influence the insertion/fixation dynamics of distinct transposon families in mammalian introns

**DOI:** 10.1186/gb-2006-7-12-r120

**Published:** 2006-12-20

**Authors:** Manuela Sironi, Giorgia Menozzi, Giacomo P Comi, Matteo Cereda, Rachele Cagliani, Nereo Bresolin, Uberto Pozzoli

**Affiliations:** 1 Scientific Institute IRCCS E Medea, Bioinformatic Lab, Via don L Monza, 23842 Bosisio Parini (LC), Italy; 2 Dino Ferrari Centre, Department of Neurological Sciences, University of Milan, IRCCS Ospedale Maggiore Policlinico, Mangiagalli and Regina Elena Foundation, 20100 Milan, Italy

## Abstract

**Background:**

Transposable elements (TEs) represent more than 45% of the human and mouse genomes. Both parasitic and mutualistic features have been shown to apply to the host-TE relationship but a comprehensive scenario of the forces driving TE fixation within mammalian genes is still missing.

**Results:**

We show that intronic multispecies conserved sequences (MCSs) have been affecting TE integration frequency over time. We verify that a selective economizing pressure has been acting on TEs to decrease their frequency in highly expressed genes. After correcting for GC content, MCS density and intron size, we identified TE-enriched and TE-depleted gene categories. In addition to developmental regulators and transcription factors, TE-depleted regions encompass loci that might require subtle regulation of transcript levels or precise activation timing, such as growth factors, cytokines, hormones, and genes involved in the immune response. The latter, despite having reduced frequencies of most TE types, are significantly enriched in mammalian-wide interspersed repeats (MIRs). Analysis of orthologous genes indicated that MIR over-representation also occurs in dog and opossum immune response genes, suggesting, given the partially independent origin of MIR sequences in eutheria and metatheria, the evolutionary conservation of a specific function for MIRs located in these loci. Consistently, the core MIR sequence is over-represented in defense response genes compared to the background intronic frequency.

**Conclusion:**

Our data indicate that gene function, expression level, and sequence conservation influence TE insertion/fixation in mammalian introns. Moreover, we provide the first report showing that a specific TE family is evolutionarily associated with a gene function category.

## Abstract 2

An analysis of humans and mouse genomes indicates that gene function, expression level, and sequence conservation influence transposable elements insertion/fixation in mammalian introns.

## Background

It is widely recognized that a large fraction of mammalian genomic DNA is accounted for by interspersed repeated elements. These sequences have been estimated to represent more than 50% of the human genome [1]. In particular, the great majority of human interspersed repeats derive from transposable elements (TEs). Four major classes of mammalian TEs have been identified in mammals: long interspersed elements (LINEs), short interspersed elements (SINEs), LTR retrotrasposons and DNA transposons.

Overall, TEs cover more than 45% of the human genome [1] but, most probably, another huge portion of human DNA is accounted for by ancient transposons that have diverged too far to be recognized as such. Indeed, different TE subtypes have been active over different evolutionary periods [2], implying that multiple copies of propagating elements accumulated over discrete time periods depending on the presence of an active source. The result of this age-dependent accumulation is that many TEs are restricted to closely related species: about a half of human repeats cannot be identified in genomes of other than primate origin [3]; similarly, most repeats that can be detected in mouse DNA are specific to rodents. Nonetheless, repeated sequences that are common to all mammalian genomes exist as they probably amplified before the mammalian radiation [3].

Once considered as merely junk DNA, it is now widely recognized that interspersed repeats have been playing a major role in genome structure evolution as well as having an impact on increased protein variability [2,4-8] and gene regulation [9]. Also, recent evidence has suggested that LINE elements have been influencing genome-wide regulation of gene expression [10] and possibly imprinting [11], while several reports [12-16] showed that specific TEs in noncoding DNA regions have been actively preserved among multiple species during evolution. Still, these observations do not contradict the 'selfish DNA' concept, regarding TEs as parasitic elements that rely more on their replication efficiency than on providing selective advantage to their host [17-19]; rather, evidence of selective benefits offered by TEs indicate that these elements have, in some instances, been 'domesticated' [20] or recruited to serve their host, a process also referred to as exaptation [21]. Several studies have suggested that TE integrations have been subjected to purifying selection to limit the genetic load imposed on their host. For example, genetic damage caused by LINE retrotransposition and ectopic recombination has been hypothesized to be responsible for selection against these elements within human loci [22]. Also, LINE and LTR elements have been reported to be underrepresented in proximity to and within genes [23], probably as a cause of their interference with regulatory processes.

In mammals the great majority of genes are interrupted by introns that usually outsize coding sequences by several fold. Similar to TEs, intervening regions were initially regarded as scrap DNA before being recognized as fundamental elements in the evolution of living organisms. TEs are abundant within intronic regions as well as in 5' and 3' intergenic spacers; yet, a comprehensive analysis of the forces driving TE insertion, fixation and maintenance within mammalian genes has still not been carried out. Here we show that gene features such as sequence conservation, function and expression level shape TE representation in human genes. Interestingly, we found evidence that a subset of loci involved in immune responses are enriched with MIR sequences; analysis of opossum orthologous genes, as well as of MIR frequency profiles, indicated that these TEs might serve a specific function in these loci.

## Results

### TE distribution varies with gene class or function

We wished to verify whether different TE types might be differentially represented depending on gene function. TE frequency varies with intron length [24] and GC percentage [1]. Moreover, in line with previous findings [24], we show that, although differences exist depending on MCS and TE age, conserved sequences have an overall negative effect on TE fixation frequency (Additional data file 1). For each TE type we therefore performed multiple regression analysis on TE number using intronic GC percentage, intron length and conserved sequence length as independent variables. The fitted values were then used to predict the expected TE number per intron (nTEi_exp_). For each gene, the TE normalized abundance (Te_na_) was calculated as follows:

(∑i∈genenTEiobs−∑i∈genenTEiexp⁡)(∑i∈genenTEiobs−∑i∈genenTEiexp⁡)
 MathType@MTEF@5@5@+=feaafiart1ev1aaatCvAUfeBSjuyZL2yd9gzLbvyNv2Caerbhv2BYDwAHbqedmvETj2BSbqee0evGueE0jxyaibaiKI8=vI8tuQ8FMI8Gi=hEeeu0xXdbba9frFj0=OqFfea0dXdd9vqai=hGuQ8kuc9pgc9s8qqaq=dirpe0xb9q8qiLsFr0=vr0=vr0dc8meaabaqaciGacaGaaeqabaqadeqadaaakeaadaWcaaqaamaabmaabaWaaabuaeaacaWGUbGaamivaiaadweacaWGPbWaaSbaaSqaaiaad+gacaWGIbGaam4CaaqabaaabaGaamyAaiabgIGiolaadEgacaWGLbGaamOBaiaadwgaaeqaniabggHiLdGccqGHsisldaaeqbqaaiaad6gacaWGubGaamyraiaadMgadaWgaaWcbaGaciyzaiaacIhacaGGWbaabeaaaeaacaWGPbGaeyicI4Saam4zaiaadwgacaWGUbGaamyzaaqab0GaeyyeIuoaaOGaayjkaiaawMcaaaqaamaabmaabaWaaabuaeaacaWGUbGaamivaiaadweacaWGPbWaaSbaaSqaaiaad+gacaWGIbGaam4CaaqabaaabaGaamyAaiabgIGiolaadEgacaWGLbGaamOBaiaadwgaaeqaniabggHiLdGccqGHsisldaaeqbqaaiaad6gacaWGubGaamyraiaadMgadaWgaaWcbaGaciyzaiaacIhacaGGWbaabeaaaeaacaWGPbGaeyicI4Saam4zaiaadwgacaWGUbGaamyzaaqab0GaeyyeIuoaaOGaayjkaiaawMcaaaaaaaa@7361@

where nTEi_obs _is the observed number of TEs per intron. These calculations were performed for all TE families in both human and mouse.

For each TE family, genes displaying three times more or less TE than expected (TE_na _> 0.5 or TE_na _< -0.5) were classified as TE-rich or TE-poor, respectively.

We next used GeneMerge [25] to retrieve significant associations; database annotations for the three categories designated by the Gene Ontology (GO) Consortium (molecular function, biological process and cellular component) were employed. Correction for multiple tests was applied to all statistical analyses. For each significant GO term retrieved, genes that are present in the study set and associate (therefore contribute) to the term are designated as 'contributing genes'. We also calculated MCS density and intergenic TE frequency of contributing genes. In particular, for intergenic sequences, TE_na _(igTE_na_) was calculated as described for introns; for contributing gene sets the fractional igTE_na _deviation was then calculated as:

(*Mean igTE*_*na *_*in contributing genes *- *mean igTE*_*na *_*in all genes*)/|*mean igTE*_*na *_*in all genes*|

Similarly, fractional MCS density deviation was calculated for contributing gene sets.

Data concerning significant (Bonferroni-corrected *p *value < 0.01) GO associations are summarized in Table 1. Three main molecular function categories were found to be associated with genes displaying low TE_na _(for more than one TE family). The first one is accounted for by genes involved in nucleic acid binding and transcription; these loci have, on average, high intronic MCS densities and few TEs in their flanking regions. The second functional category is represented by genes coding for cytokines/growth factors/hormones and, more generally, receptor ligands: these genes do not have, as a whole, higher than average intron conservation and, with the exception of LTR-poor genes, tend to have low igTE_na_. The last category (not present among Alu-poor genes) is accounted for by structural molecules, mainly represented by ribosomal proteins. These genes have extremely low MCS densities and igTE_na_. These same associations were retrieved for mouse genes (supplementary Table 1 in Additional data file 2), although no GO term was significantly associated with L1-depleted mouse genes.

Significant associations were also identified with biological process GO terms. As expected [1,26] genes involved in morphogenesis/development were over-represented in most TE-poor groups and displayed extremely conserved intronic regions as well as few intergenic TEs (except for LTRs). Also, loci involved in immune defense/response to stimulus were found to be over-represented among TE-poor genes. These loci also have less TEs in their flanking regions and, on average, low MCS densities. Consistently with molecular function GO term retrieval, genes involved in biological processes such as transcription and metabolism were found to be overrepresented among TE-poor groups. Again, similar findings were obtained when mouse genes (supplementary Table 1 in Additional data file 2) were analyzed, although no biological process GO term was significantly over-represented among genes displaying low LINE or DNA transposon frequencies.

Moreover, a relatively small set of genes involved in sexual reproduction/spermatogenesis were found to display lower than expected MIR frequencies (both in introns and intergenic sequences) in humans but not in rodents.

### TE-rich gene categories

Genes displaying higher than expected TE frequencies were also identified for all repeat families, although they were less numerous than TE-poor genes. GO analysis retrieved significant associations (Bonferroni-corrected *p *value < 0.01) only for MIR-rich human genes (Table 2).

GO terms associated with high MIR density differed between human (Table 2) and mouse (Table 3); in particular, MIR-rich genes belong to the immune response pathway in humans, while they mainly code for ion channels in mice. In both mammals, MIR density in these genes is not accounted for by fewer integrations of younger TEs since MIR frequency remains significantly higher than the average when calculated on TE-free (unique) intron size. To gain further insight into this issue, we singled out all genes contributing to at least one GO term in Table 2 (85 genes) and searched for a murine ortholog in our mouse gene dataset; 61 best unique reciprocal orthologs were identified and their MIR density (calculated on unique intron sequence) was significantly higher (Wilcoxon rank sum test, *p *< 10^-14^) than the average (calculated on all murine genes in our dataset). The same procedure was applied to mouse MIR-rich genes contributing to GO terms in Table 3; again, human genes displayed significantly higher intronic MIR densities (Wilcoxon rank sum test, *p *< 10^-14^). The difference between human and mouse in GO terms associated with MIR-rich genes, therefore, results from the cut-off we used (TE_na _> 0.5, corresponding to three times more than expected) to define MIR-rich genes.

We next wished to verify whether these genes also had higher frequencies of other ancestral TEs, namely L2s and DNA transposons. The frequencies of these elements were calculated on TE-free intron size and no significant differences were identified in either human or mouse when MIR-rich genes involved in immune responses were compared to all genes (not shown); this finding suggests that relaxation of selective constraints allowing accumulation of ancestral TE insertions is not responsible for MIR over-representation in these genes. Conversely, MIR-rich ion channel introns also displayed significantly higher frequencies of both DNA transposons and L2s, indicating, therefore, that the relative enrichment in old TEs is not specific to MIRs.

**Figure 1:**
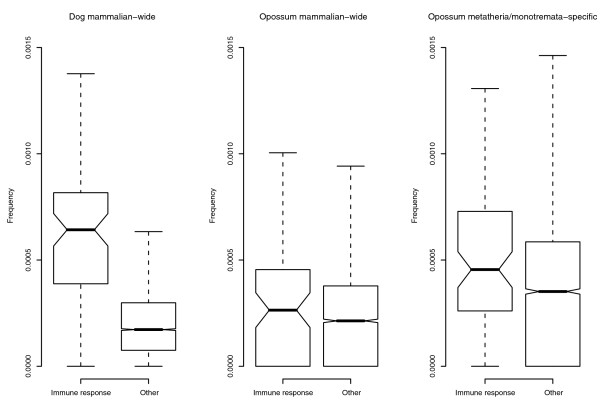
Analysis of MIR frequency in dog and opossum immune defense genes. MIR sequences were divided into mammalian-wide and metatherian/monotremata-specific. Immune response genes displayed significantly higher frequencies of both MIR types compared to the remaining genes. Box height represents sample interquartile range and the bold line depicts the median position. The whiskers extend to the most extreme data point, which is no more than 1.5 times the interquartile range from the box.

We therefore wished to verify whether high MIR frequency in immune response genes also occurs in mammalian species other than human and mouse. We therefore analyzed MIR frequency in dog, as well as in our most distant extant mammalian ancestors, namely metatherian. To this aim we searched both *Canis familiaris *and *Monodelphis domestica *(gray short-tailed opossum) annotation tables and retrieved dog/opossum genomic positions corresponding to human transcripts in our dataset. A total of 5,476 human genes could be located on the *Monodelphis *sequence (7,454 on the dog sequence) and, out of 85 MIR-rich immune response genes, 77 were identified in opossum (79 in dog). We then calculated the frequency of mammalian-wide MIRs within dog and opossum genes: in both species (Figure 1) immune response loci displayed significantly higher frequencies compared to the remaining genes (Wilcoxon rank sum test, *p *< 10^-15 ^and 0.022 for dog and opossum, respectively). Interestingly, in addition to mammalian-wide MIR sequences, metatherian/monotremata-specific MIR-related TEs are interspersed in the opossum genome. These latter are mainly accounted for by MON1 and MAR1 [3], and show 90% identity with the MIR core sequence [27]. Opossum immune response loci also display higher metatherian/monotremata-specific MIR frequencies compared to the remaining genes (Wilcoxon rank sum test, *p *= 0.0023) (Figure 1).

### Characterization of MIR sequences associated with immune response genes

**Figure 2:**
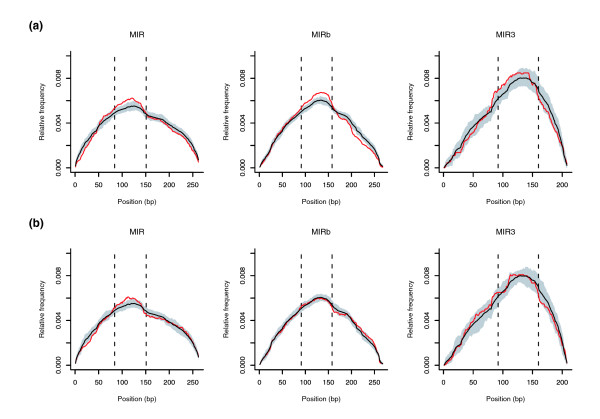
Analysis of human MIR sequences associated with immune response genes. **(a) **Relative frequency at each position of MIR (*n *= 277), MIRb (*n *= 382) and MIR3 (*n *= 104) consensus sequences in immune response gene introns (red lines). Mean profiles and intervals corresponding to the 1st and 99th percentiles in 100 random sample frequency distributions are represented by black lines and grey areas, respectively. **(b) **The same as in (a) for MIRs located in intergenic regions. MIR, *n *= 239; MIRb, *n *= 345; MIR3, *n *= 97. Hatched lines delimit the MIR CORE region.

We next wished to verify whether MIR sequences in immune response genes have some feature distinguishing them from MIRs in other genomic locations. Four highly related MIR subtypes (MIR, MIR3, MIRb and MIRm) have been identified in the murine and human genomes [3]; the four subtypes display a central, almost identical 70 base-pair (bp) core region [28]. To verify whether any MIR region has been preferentially retained in MIR-rich immune response genes, we retrieved all MIR elements located in the intronic regions of these genes or in their flanking intergenic spacers. In the latter case, we restricted the analysis to TEs located within 15 kb of 5' or 3' gene boundaries. We next used the different MIR subtype reference sequences [3] to align all instances in immune response gene introns or intergenic spacers separately. To verify whether any MIR region was over- or under-represented in these genes, we compared the average relative frequency at each position with frequencies derived from 100 samples of an equal number of MIR sequences randomly selected from either introns or intergenic spacers. The mean, as well as the 1st and 99th percentiles in random sample frequency distributions were then calculated at each position; they are plotted in Figure 2a together with average frequencies of MIRs located in immune response genes. This calculation was not performed for MIRm sequences because of their paucity (47 instances in immune genes). The frequency profile of MIR, MIR3 and MIRb sequences located in immune response gene introns indicates that the central core region is over-represented (beyond the 99th percentile) compared to the background intronic frequency. These same findings did not apply to MIRb and MIR3 sequences in intergenic regions flanking immune response genes (Figure 2b). Similar results (supplemental Figure 2 in Additional data file 2) were obtained for mouse MIR sequences located in immune response genes.

**Figure 3:**
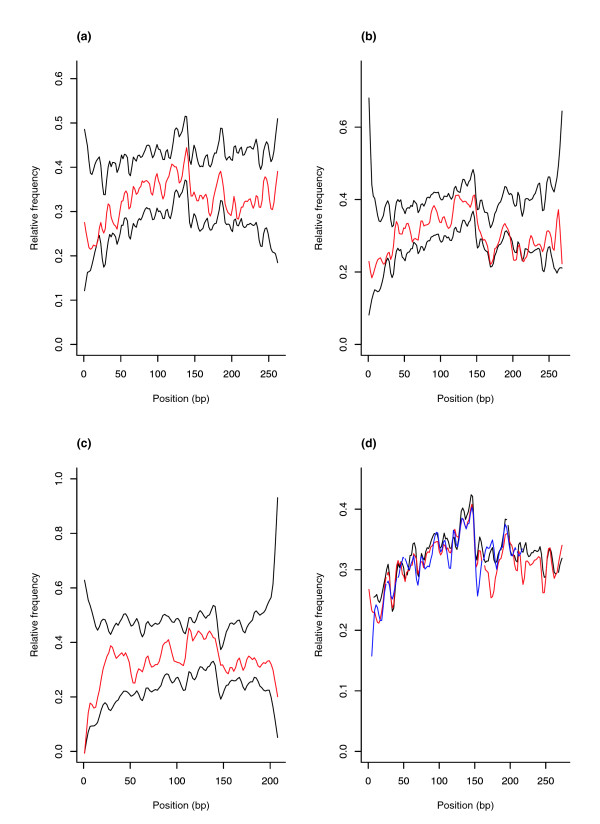
Co-conservation profile of MIR sequences. Co-conservation frequency at each position of **(a) **MIR (*n *= 277), **(b) **MIRb (*n *= 382) and **(c) **MIR3 (*n *= 104) consensus sequences in immune response gene introns (red lines). Frequency intervals corresponding to the 1st and 99th percentiles in 100 random sample frequency distributions are represented by the black lines. **(d) **Co-conservation profiles of MIR sequences located in human introns; in this case, positions correspond to the alignment of the three MIR subtypes: MIR (black), MIRb (red) and MIR3 (blue).

We therefore analyzed the human/mouse co-conservation profile (that is, the frequency of bases that, in both human and mouse, are equal to the MIR consensus sequence) of human/mouse orthologous MIR instances. No significant difference was observed (Figure 3a-c) between MIRs located in immune response introns and random MIR samples. Yet, as is evident from Figure 3d, the central portion of intronic MIR sequences, either located in defence response genes or not, is more frequently co-conserved compared to 5' and 3' flanking regions.

### Repeat content as a function of expression level

**Figure 4:**
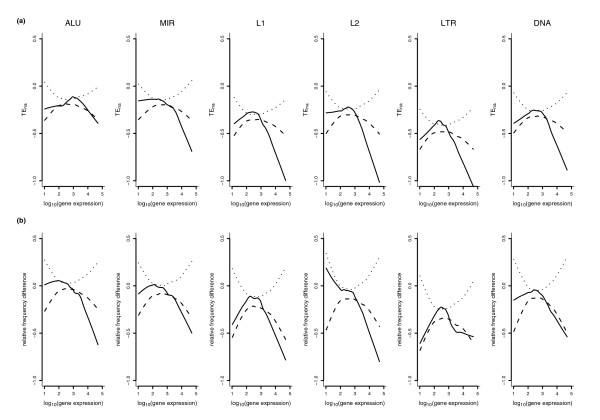
Gene-expression dependent variation in TE intronic abundance. Gene expression levels were derived from microarray data. **(a) **Lowess fit (solid line) and probability intervals (hatched lines) of TE_na _versus gene expression level (log transformed values) for the six TE families. **(b) **Lowess fit (solid line) and probability intervals (hatched lines) of intronic to intergenic relative TE frequency difference (see text) versus gene expression level (log transformed values).

Different TE types have been reported to differentially associate with gene regions depending on expression levels [29]. To get further insight into this issue, we calculated expression level (averaged over all tissues) for human and mouse genes in our dataset. Since different experimental methods for measuring gene expression have been shown to yield different results [30], we used expression data derived from two different experimental methods, namely microarray and serial analysis of gene expression (SAGE). For each family, TE_na _was then plotted against expression level and lowess curves calculated (see Materials and methods for details). To address the significance of the observed trends, 100 lowess smooths were calculated after random data permutations and empirical probability intervals were calculated (see Materials and methods). As is evident from Figure 4a, a marked decrease in TE_na _is observed for genes above the 70th to 80th gene expression percentile. Results obtained from SAGE expression data, as well as for murine genes, gave similar results and are available in Additional data file 2.

To gain further insight, we wished to compare intronic with intergenic TE frequecies (TE number/sequence length). In fact, intergenic and intronic regions belong to the same isochore (that is, they display a similar CG percentage) and their lengths are correlated [31], as well as their MCS density (Spearman rho = 0.37, *p *< 10^-16^); therefore, TE density can be directly compared. Thus, for each gene we calculated the relative frequency difference as:

(*TEf*_*intron*_/*meanTEf*_*intron*_) - (*TEf*_*inter*_/*meanTEf*_*inter*_)

where *TEf*_*intron *_is the average TE frequency for all introns in the same gene, *meanTEf*_*intron *_is the average TE frequency for all introns in all genes, *TEf*_*inter *_is the TE frequency averaged for 5' and 3' regions flanking each gene and *meanTEf*_*inter *_is the average TE frequency for all intergenic spacers. Again lowess curves were obtained, as well as empirical probability intervals derived from 100 random permutations; as shown in Figure 4b, for highly expressed genes and for all TE types, a significant decreasing trend is observed when frequency differences are plotted against gene expression. The same observations were confirmed using expression data derived from SAGE experiments and they also apply to mouse genes (supplementary Figures 3 to 5 in Additional data file 2). It is worth noting that very similar results were also obtained when the same calculations were performed using 8 kb sequences flanking each gene (4 kb each side) instead of entire intergenic regions (supplementary Figure 6a,b in Additional data file 2 for human genes and data obtained with either microarray or SAGE, respectively). For the latter analyses only genes displaying both 3' and 5' intergenic regions longer than 10 kb were selected (*n *= 3,477).

## Discussion

TE distribution in mammalian genomes has been addressed in numerous studies. Yet, many questions concerning the nature of the host-element relationship still remain unanswered and a comprehensive scenario of the selective forces affecting TE fixation in mammalian genomes is still missing. In particular, genome-wide analyses of TE type distribution within and in proximity to human genes have often neglected relevant features, such as sequence conservation, gene function and expression level.

Since the precise removal of an inserted transposon is a rare event [32], present day TE distribution is the result of insertion frequency and fixation probability over time. Previous work had indicated that TE frequency inversely correlates with different measures of noncoding sequence conservation [24,33,34]. We confirm here (see Additional data file 1) that these observations are explained by the intrinsic mutagenic potential of transposition and the necessity of preserving multispecies conserved sequences from disruption. In fact, TE insertion is counterselected at different degrees depending on the relative timing of MCS fixation and TE activity. Given this premise and considering insertion to be mutagenic irrespective of TE family or type, we analyzed the distribution of different TEs in human introns after correcting for the known parameters affecting either integration frequency or fixation probability, namely GC content [1,35], intron size [24,34] and MCS density (this study and [24]). All analyses have been carried out in parallel on human and mouse genes. Such a procedure strengthens the ensuing conclusions since the majority of TEs are specific to either species [3] and the maintenance of ancestral TEs also differs between primates and rodents due to the higher mutation rate of the latter [34]. Also, we analyzed intronic TE distribution in association with both MCS content and TE abundance in intergenic regions. In fact, although we corrected for MCS presence in multiple regression fitting, MCS content represents an indication of gene complexity and regulatory accuracy [36]. On the other hand, TE representation in intergenic spacers might highlight differences in TE effect depending on location; this is especially relevant for TE families that have been previously reported to be preferentially abundant in intergenic versus intronic regions or *vice versa *[23].

The initial analysis of the human genome sequence [1] had indicated that the *HOX *gene cluster is virtually deprived of TEs; the same result was obtained upon analysis of the mouse genome and interpreted in terms of TEs disturbing fine tuned regulation of developmental genes. A more recent study indicated that TE-free regions are significantly associated with genes coding for developmental regulators or transcription factors [26].

Our GO data indicate that functional classes associated with TE-poor genes extend well beyond highly conserved gene categories such as developmental regulators and transcription factors. In fact, some MCS-poor gene function categories also display lower than expected TEs; genes coding for structural molecules and ribosomal proteins are deprived of most TE families in both introns and intergenic spacers. These loci are mainly accounted for by housekeeping genes; if low TE representation in intronic regions might be explained by the need to reduce transcriptional costs (in agreement with TE paucity in introns of highly expressed genes, as discussed below), the reason why TEs are also excluded from intergenic spacers is more difficult to explain. One possibility is that extensive methylation of repetitive elements might exert a negative regulation on nearby gene expression with detrimental consequences for housekeeping genes. Indeed, several reports [37-40] have suggested the existence of specific methylation patterns in TEs (probably representing a cellular defence mechanism against transposition) and methylation has been shown to spread in *cis *from TEs to flanking cellular sequences in plants and yeast [41,42]. In this respect, it is intriguing that Alus, which show lower methylation levels [40], possibly due to their association with a 'protective' sperm protein [43], are not preferentially excluded from these same housekeeping gene sets (Table 1). Similar considerations might be applied to genes coding for cytokines, growth factors, and hormones as well as genes involved in immune responses, all of which display few intronic and intergenic TEs. Still, these genes are not housekeeping genes or highly expressed and they also display lower than expected Alu frequencies. We speculate that these gene categories might require extremely subtle regulation of transcript levels (especially in the case of secreted proteins) or precise timing of activation (for example, in response to a stimulus). Indeed, altered hormone or cytokine levels have been associated with human disease and cancer (reviewed in [44,45]), while the effects of immune response gene misregulation are easily envisaged. As mentioned above, TEs can influence gene expression by both altering the epigenetic state of TE-carrying alleles [46,47] and providing promoters and transcription factor binding sites (either enhancers or suppressors (reviewed in [48,49]) to the genes neighboring their integration sites. In particular, Alus have been shown to potentially carry functional sites for different transcription factors as well as for both steroid-hormone and retinoic acid receptors (reviewed in [48]); these observations have led to the speculation that Alu integration might cause a genetic disease not through gene coding sequence disruption but rather through alteration of gene expression patterns [50]. Indeed, several gene categories displaying lower than expected intronic Alu frequencies also show significantly fewer Alus in flanking intrergenic spacers.

It is interesting to notice that genes involved in immune response, which display extremely low conservation in both coding [51-53] and non-coding sequences [36], as well as a higher content of TEs in their untranslated sequences [54], are deprived of most TE types but enriched in MIR sequences in three eutherian species (human, mouse and dog). Given the partially independent origin of MIR sequences in eutheria and metatheria, it is important to notice that analysis of orthologous genes indicated that MIR over-representation also occurs in opossum immune response genes, suggesting the evolutionary conservation of a specific function for MIRs located in these loci.

MIRs belong to a large TE superfamily referred to as CORE-SINE [53]; all CORE-SINE TEs share a common 65 bp central region that was proposed to be either relevant for retrotranspositional activity [27,55] or functional in the host genome [28]. Previous studies noted a higher representation in mammalian genomes of MIR core regions compared to flanking 3' and 5' sequences [12,28]; our data indicate that the core sequence is both more frequent and more conserved in the human genome, as assessed by co-conservation profiles. Since MIRs are thought to be long time fossils [28], this observation suggests that the core might serve some general function in mammalian genomes. Indeed, upon analysis of aligned human-mouse intergenic sequences, Silva *et al*. [12] suggested that the core region is more often present in aligning orthologous regions than expected on the basis of background genome frequency. Our data indicate that this observation also applies to MIR sequences located in immune response gene introns. To our knowledge, this is the first report showing that a specific TE family is evolutionarily associated with a gene function category. Whether MIRs located in defense response genes serve a specific function or they share a common role with the other core sequences in the genome remains to be elucidated. Recent works indicated that two ancient SINE families have been extensively exapted in the human genome and copies of these TEs have been recruited to serve distinct functions in different genomic locations [14,16]. This might also be the case for MIRs; alternatively, these sequences might all share a general role in the human genome that is particularly important in immune defense loci.

The last part of our work is devoted to studying the influence of gene expression level on TE distribution. In fact, despite the small population size, it has been reported that human genes show signatures consistent with selection mediated by expression levels [56]. In particular, selective pressure aimed at reducing transcriptional cost has been proposed to act on highly expressed human genes and TEs had been suggested as possible targets for selection to act upon [57]. Our findings strongly support this view: all TE families are under-represented in highly expressed genes. While the ability of LINE L1s to affect mRNA transcription/processing efficiency [10] might explain their exclusion from highly expressed introns, Alus have been reported to associate with highly expressed gene regions [29] and no direct effect on transcription or processing has ever been described for ancestral TE families. Therefore, the expression-dependent exclusion of all TE families from intronic regions is strongly consistent with the need to reduce the transcription energetic costs. The issue had also been raised as to whether a selective pressure is still acting on highly expressed genes or if we merely witness the remnants of a previous action of selection (still not at equilibrium) [56]. Our data support the first hypothesis: Alus, which represent relatively young TEs are under-represented in highly expressed introns and, in both human and mouse, separation in TE divergence classes did not reveal any different expression-dependent association with TE age (not shown).

## Materials and methods

### Sequence retrieval and analysis

For creation of the intron database, human genes that had been annotated in the NCBI Reference Sequence (RefSeq) collection were selected (reviewed or validated entries only); for mouse genes 'Provisional' entries were also included. Genomic sequences, intron/exon boundaries and intergenic regions were derived from the UCSC genome annotation database [58] (assembly hg17 for human and mm5 for mouse). Intronless genes were discarded and, for each gene, the transcript corresponding to the longest genomic sequence and containing the highest number of exons was selected. The datasets are composed of 7,614 human and 5,550 mouse genes, accounting for 81,599 and 55,553 introns, respectively. For each gene, the closest 5' and 3' known genes were identified (using the UCSC knownGene table [58]); intergenic regions were defined as the genomic portions extending upstream and downstream of the transcribed region to the closest gene.

Transposable elements were identified and categorized using the UCSC annotation tables that rely on RepeatMasker. MCS were obtained using phastCons predictions [13,59], which are based on a phylogenetic hidden Markov model and are available through the UCSC database (phastConsElements Table [58]). MCSs were derived from human/chimpanzee/mouse/rat/dog/chicken/pufferfish/zebrafish multiz alignments [58].

Only purely noncoding phastCons elements were selected (that is, MCSs partially overlapping with exons were discarded); a total of 238,005 and 596,018 human MCSs were retrieved in introns and intergenic sequences, respectively. In mouse, 133,458 intronic and 312,752 intergenic MCSs were identified.

For the identification of human-mouse orthologous pairs, the EnsMart database [60] was interrogated and only entries representing unique best reciprocal hits were selected.

Retrieval of opossum and dog genes and annotations was performed using UCSC tables [58] referring to assembly monDom1 and canFam2. In particular, opossum/dog mRNA accession numbers were identified by cross-referencing tables 'geneName' and 'gbCdnaInfo' [58]. Genomic locations were next retrieved through tables xenoMrna or blastHg17KG. *Monodelphis *and dog TE annotations were directly obtained from UCSC [58] and MIR number per gene was calculated as the number of distinct elements fully contained between gene boundaries. MIR frequency was calculated as MIR number/gene length.

### Gene classification

Gene associations with GO terms and their descriptions were performed by cross-referencing the UCSC hg17 kgXref table [57] with the GO database [61]. Association and description files were then created and significant associations between gene groups and GO terms were identified using GeneMerge [25].

### MIR sequence analysis

MIR consensus sequences were derived from the Genetic Information Research Institute (RepeatMasker database, release 20060314) [62].

For calculation of the MIR relative frequency profile, human or mouse MIR instances were aligned to the consensus sequence using SWAT [63]. Microinsertions in human and mouse instances were ignored. The relative frequency profile at each position of the consensus sequence was calculated as the number of instances covering the position divided by the total number of bases in instances.

For calculation of MIR co-conservation profiles, we used the liftOver utility at the UCSC genome browser [58] to obtain human/mouse orthologous MIR instances. MIRs were then aligned to the reference sequence using ClustalW. Microinsertions in human instances were ignored. For each MIR position in human instances we calculated the frequency of co-conservation (that is, the frequency of bases that, in both human and mouse, are equal to the MIR consensus sequence). This procedure was applied to both MIRs located in immune response introns and to 100 randomly selected MIR samples of equal size and located within intronic regions. The co-conservation profile was then calculated using a smoothing spline with a span of ten bases over non-CpG positions.

### Expression data

Microarray expression data for human and mouse genes were derived from previous high-throughoutput gene expression studies [64,65]; they are publicly accessible through the UCSC database (tables 'gnfHumanAtlas2median' and 'gnfHumanAtlas2medianExps', and 'gnfMouseAtlas2median' and 'gnfMouseAtlas2medianExps') [58]. We only considered probes corresponding to genes that had been included in our database; signals from duplicated probes on the same chip were averaged as well as replicates from the same tissue. A gene was considered to be expressed in a given tissue if its signal level was higher or equal to 200 arbitrary units, as previously recommended [64]. Data derived from tumor tissues were discarded. In the case of SAGE data, for each transcript entry in our databases we extracted a SAGE tag (10 bp downstream of the most 3' *Nla*III site). For both human and mouse, tags were then matched to all RefSeq mRNAs and purged if they corresponded to more than one transcript.

SAGE libraries were obtained from the SAGE Genie website [66]; for both organisms, libraries containing less than 20,000 tags, corresponding to tumor tissues, uncharacterized tissues, pharmacological treatments and mutated samples were discarded. As previously suggested [67], libraries with mean tag GC content >0.5 were also removed. We retained 81 human libraries (both long and short tags), accounting for 21 tissues; for mouse, we retained 98 libraries accounting for 41 tissues.

Finally, we added all counts for libraries representing the same tissue type and converted absolute tag counts to relative tag counts (counts per million).

### Statistical analysis

All statistical analyses were performed using R [68]. Locally weighted scatter plot smoothing was performed using lowess curves [69]. These curves are produced by weighted least-square linear fitting within a window sliding through the data. The size of the window (span) controls the degree of smoothing and the curves are made robust by iterating the fit within each window discarding outliers. In all cases 5 robustifying iterations were performed and a span of 0.5 was used. To allow empirical *p *value calculations, we performed 100 independent random data permutations of the variable on the y axis. Indeed, computing lowess smooths after random permutations of the data can be used as a reference to gauge the significance of the pattern observed on the actual data [70].

Probability interval limits were chosen, for each x value, as corresponding to *p *= 0.005 and *p *= 0.995 in the distribution of the 100 permutated y values considered as a Gaussian.

## Additional data files

The following additional data are available with the online version of this paper. Additional data file 1 contains supplementary text presenting analysis of MCS density and TE integration frequency over evolutionary time. A supplementary figure describing the results is also provided (supplementary Figure 1) together with its legend. Additional data file 2 contains supplementary Table 1, and supplementary Figures 2 to 6 and their legends: supplementary Table 1 lists GO terms associated with mouse TE-poor genes; supplementary Figure 2 shows analysis of murine MIR sequences associated with immune response genes; supplementary Figure 3 shows gene-expression dependent variation in TE intronic abundance for human genes (SAGE data); supplementary Figure 4 shows gene-expression dependent variation in TE intronic abundance for mouse genes (microarray data); supplementary Figure 5 shows gene-expression dependent variation in TE intronic abundance for mouse genes (SAGE data); supplementary Figure 6 shows intronic to intergenic relative frequency difference (calculated on gene flanks rather than entire intergenic regions).

## Supplementary Material

Additional data file 1 Supplementary text presenting analysis of MCS density and TE integration frequency over evolutionary time. A supplementary figure describing the results is also provided (supplementary Figure 1) together with its legend. Click here for file

Additional data file 2 Supplementary Table 1 lists GO terms associated with mouse TE-poor genes. Supplementary Figure 2 shows analysis of murine MIR sequences associated with immune response genes. Supplementary Figure 3 shows gene-expression dependent variation in TE intronic abundance for human genes (SAGE data). Supplementary Figure 4 shows gene-expression dependent variation in TE intronic abundance for mouse genes (microarray data). Supplementary Figure 5 shows gene-expression dependent variation in TE intronic abundance for mouse genes (SAGE data). Supplementary Figure 6 shows intronic to intergenic relative frequency difference (calculated on gene flanks rather than entire intergenic regions). Click here for file

## Figures and Tables

**Table 1:** GO terms associated with TE-poor genes

		Under-represented TE type																	
		
GO term	Description	Alu			L1			L2			LTR			DNA transp.			MIR		
	**Molecular function**	N	MCS	IG	N	MCS	IG	N	MCS	IG	N	MCS	IG	N	MCS	IG	N	MCS	IG

GO:0003676	Nucleic acid binding	-	-	-	-	-	-	468	0.88*	-0.44*	598	0.86*	-0.27*	-	-	-	327	1.07	-0.29*
GO:0003677	DNA binding	-	-	-	-	-	-	-	-	-	394	1.27*	-0.17	-	-	-	219	1.6*	-0.34*
GO:0003723	RNA binding	-	-	-	-	-	-	131	0.08	-0.49*	153	0.13	-0.42*	-	-	-	91	0.03	-0.12
GO:0003700	Transcription factor activity	138	2.45*	-0.63*	171	1.9*	-0.51*	160	2.1*	-0.41*	220	1.82*	-0.09	165	2.18*	-0.65*	125	2.23*	-0.76*
GO:0030528	Transcription regulator activity	159	2.35*	-0.59*	-	-	-	-	-	-	279	1.57*	-0.1	-	-	-	152	2.04*	-0.67*
GO:0004871	Signal transducer activity	348	0.32	-0.45*	-	-	-	-	-	-	-	-	-	-	-	-	-	-	-
GO:0004888	Transmembrane receptor activity	138	0.23	-0.31	-	-	-	-	-	-	-	-	-	-	-	-	-	-	-
GO:0005102	Receptor binding	137	0.5	-0.57*	170	0.29	0.03	149	0.24	0.14	192	0.33	0.2	155	0.32	-0.02	-	-	-
GO:0001664	G-protein-coupled receptor binding	-	-	-	25	-0.14	-0.23	-	-	-	-	-	-	26	-0.16	-0.1	-	-	-
GO:0008083	Growth factor activity	47	0.98	-0.16	-	-	-	-	-	-	64	0.73	0.45*	-	-	-	-	-	-
GO:0005125	Cytokine activity	69	0.59	-0.71*	84	0.29	-0.36	-	-	-	91	0.44	0.48*	76	0.42	0.24	-	-	-
GO:0008009	Chemokine activity	-	-	-	25	-0.14	-0.23	-	-	-	-	-	-	26	-0.16	-0.1	-	-	-
GO:0042379	Chemokine receptor binding	-	-	-	25	-0.14	-0.23	-	-	-	-	-	-	26	-0.16	-0.1	-	-	-
GO:0005179	Hormone activity	33	0.49	-0.71	-	-	-	41	0.11*	-0.44	-	-	-	34	0.19*	-0.47	27	0.49	-0.64
GO:0005184	Neuropeptide hormone activity	10	-0.12	0.27	-	-	-	11	0.01	0.68	-	-	-	-	-	-	-	-	-
GO:0004252	Serine-type endopeptidase activity	-	-	-	50	-0.34*	-0.01	-	-	-	-	-	-	-	-	-	-	-	-
GO:0004263	Chymotrypsin activity	-	-	-	38	-0.45*	-0.1	-	-	-	-	-	-	-	-	-	-	-	-
GO:0004295	Trypsin activity	-	-	-	39	-0.45*	-0.21	-	-	-	-	-	-	-	-	-	-	-	-
GO:0003735	Structural constituent of ribosome	-	-	-	100	-0.34*	-0.25	89	-0.41*	-0.72*	116	-0.37*	-0.58*	79	-0.35*	-0.5	63	-0.33*	-0.47
GO:0005198	Structural molecule activity	-	-	-	212	-0.04	-0.4*	192	-0.11*	-0.43*	260	-0.07	-0.2	-	-	-	-	-	-
	**Biological process**																		
GO:0007275	Development	335	1.41*	-0.55*	410	1.13*	-0.45*	386	1.19*	-0.23	512	1.09*	0.1	384	1.32*	-0.45*	258	1.58*	-0.48*
GO:0009653	Morphogenesis	222	1.24*	-0.48*	-	-	-	-	-	-	334	0.94*	0.21*	-	-	-	-	-	-
GO:0009887	Organogenesis	186	1.03*	-0.46*	-	-	-	-	-	-	270	0.8*	0.22*	-	-	-	-	-	-
GO:0009888	Histogenesis	-	-	-	-	-	-	-	-	-	47	0.49	0.46	-	-	-	-	-	-
GO:0008544	Epidermis development	24	-0.27	-1.4*	-	-	-	-	-	-	-	-	-	-	-	-	-	-	-
GO:0001501	Skeletal development	36	1.4*	-0.23	-	-	-	-	-	-	-	-	-	-	-	-	-	-	-
GO:0007267	Cell-cell signaling	137	0.71*	-0.27	162	0.69*	0.03	-	-	-	-	-	-	-	-	-	-	-	-
GO:0007166	Cell surface receptor linked signal transduction	161	0.29*	-0.45*	-	-	-	-	-	-	-	-	-	-	-	-	-	-	-
GO:0007186	G-protein coupled receptor protein signaling pathway	93	0.17	-0.51*	-	-	-	-	-	-	-	-	-	-	-	-	-	-	-
GO:0006952	Defense response	172	0.13*	-0.75*	217	-0.08*	-0.16	202	-0.11*	-0.19	259	0*	0.01	219	-0.04*	-0.2	-	-	-
GO:0006955	Immune response	155	0.17*	-0.7*	201	-0.08*	-0.19	-	-	-	-	-	-	202	-0.05*	-0.17	-	-	-
GO:0050896	Response to stimulus	268	0.13	-0.61*	-	-	-	-	-	-	-	-	-	-	-	-	-	-	-
GO:0009607	Response to biotic stimulus	187	0.1*	-0.69*	240	-0.1*	-0.21	222	-0.14*	-0.16	290	-0.05*	0	235	-0.07*	-0.25	-	-	-
GO:0009613	Response to pest, pathogen or parasite	99	-0.02*	-0.8*	-	-	-	-	-	-	-	-	-	127	-0.33*	-0.13	-	-	-
GO:0043207	Response to external biotic stimulus	106	-0.09*	-0.86*	-	-	-	-	-	-	-	-	-	134	-0.36*	-0.17	-	-	-
GO:0006817	Phosphate transport	27	-0.05	-0.39	-	-	-	-	-	-	-	-	-	-	-	-	-	-	-
GO:0006820	Anion transport	41	0.03	-0.47	-	-	-	-	-	-	-	-	-	-	-	-	-	-	-
GO:0015698	Inorganic anion transport	38	0.03	-0.49	-	-	-	-	-	-	-	-	-	-	-	-	-	-	-
GO:0006350	Transcription	-	-	-	-	-	-	-	-	-	386	1.22*	-0.16	-	-	-	211	1.43*	-0.43*
GO:0045449	Regulation of transcription	-	-	-	-	-	-	-	-	-	365	1.31*	-0.15	-	-	-	198	1.53*	-0.45*
GO:0006351	Transcription, DNA-dependent	-	-	-	-	-	-	-	-	-	369	1.25*	-0.16	-	-	-	203	1.48*	-0.45*
GO:0006355	Regulation of transcription, DNA-dependent	-	-	-	-	-	-	267	1.38*	-0.23	355	1.31*	-0.16	-	-	-	196	1.53*	-0.46*
GO:0006139	Nucleobase, nucleoside, nucleotide and nucleic acid metabolism	-	-	-	-	-	-	-	-	-	-	-	-	-	-	-	301	1.08	-0.23
GO:0019219	Regulation of nucleobase, nucleoside, nucleotide and nucleic acid metabolism	-	-	-	-	-	-	-	-	-	371	1.29*	-0.16	-	-	-	202	1.51*	-0.46*
GO:0019222	Regulation of metabolism	-	-	-	-	-	-	303	1.32*	-0.2	409	1.24*	-0.16	-	-	-	217	1.54*	-0.38*
GO:0006412	Protein biosynthesis	-	-	-	144	-0.14	-0.34	-	-	-	179	-0.1*	-0.48*	-	-	-	-	-	-
GO:0050876	Reproductive physiological process	18	1.19	-0.76	-	-	-	-	-	-	-	-	-	-	-	-	-	-	-
GO:0000003	Reproduction	-	-	-	-	-	-	-	-	-	-	-	-	-	-	-	44	0.09*	-0.38
GO:0019953	Sexual reproduction	-	-	-	-	-	-	-	-	-	-	-	-	-	-	-	43	0.06*	-0.38
GO:0007276	Gametogenesis	-	-	-	-	-	-	-	-	-	-	-	-	-	-	-	39	0.14*	-0.39
GO:0048232	Male gamete generation	-	-	-	-	-	-	-	-	-	-	-	-	-	-	-	33	0.07*	-0.05
GO:0007283	Spermatogenesis	-	-	-	-	-	-	-	-	-	-	-	-	-	-	-	33	0.07*	-0.05

Significant differences are marked with an asterisk. DNA transp., DNA transposon; N, number of contributing genes; MCS, fractional intronic MCS density deviation (see text); IG, fractional igTEna deviation (see text).

**Table 2:** GO terms associated with TE-rich human genes

		Over-represented TE types							
GO term	Description	Alu	L1	L2	LTR	DNA transp.	MIR		

	**Molecular function**						N	MCS	IG

GO:0008009	Chemokine activity	-	-	-	-	-	9	-0.91*	-0.66
GO:0005125	Cytokine activity	-	-	-	-	-	24	-0.42	-0.13
GO:0001584	Rhodopsin-like receptor activity	-	-	-	-	-	19	-0.44	0.31
GO:0042379	Chemokine receptor binding	-	-	-	-	-	9	-0.91*	-0.66
GO:0005102	Receptor binding	-	-	-	-	-	38	-0.45	-0.03
GO:0001664	G-protein-coupled receptor binding	-	-	-	-	-	9	-0.91*	-0.66
	**Biological process**								
GO:0050874	Organismal physiological process	-	-	-	-	-	89	-0.57*	0.01
GO:0009607	Response to biotic stimulus	-	-	-	-	-	70	-0.69*	0.36
GO:0006955	Immune response	-	-	-	-	-	60	-0.67*	0.23
GO:0009611	Response to wounding	-	-	-	-	-	31	-0.73*	0.11
GO:0006954	Inflammatory response	-	-	-	-	-	24	-0.79*	0.06
GO:0006952	Defense response	-	-	-	-	-	66	-0.7*	0.3
GO:0045087	Innate immune response	-	-	-	-	-	26	-0.78*	0.07
GO:0016064	Humoral defense mechanism	-	-	-	-	-	14	-0.65	0.24
GO:0009617	Response to bacteria	-	-	-	-	-	13	-0.83*	0.34
GO:0009613	Response to pest, pathogen or parasite	-	-	-	-	-	47	-0.72*	0.21
GO:0043207	Response to external biotic stimulus	-	-	-	-	-	51	-0.74*	0.16
GO:0006950	Response to stress	-	-	-	-	-	53	-0.72*	0.16
GO:0042742	Defense response to bacteria	-	-	-	-	-	9	-0.98*	0.36
GO:0009605	Response to external stimulus	-	-	-	-	-	65	-0.76*	0.19
GO:0009620	Response to fungi	-	-	-	-	-	6	-1*	0.91
GO:0009628	Response to abiotic stimulus	-	-	-	-	-	28	-0.83*	0.55
GO:0042221	Response to chemical substance	-	-	-	-	-	27	-0.83*	0.7
GO:0050896	Response to stimulus	-	-	-	-	-	85	-0.71*	0.31
GO:0006968	Cellular defense response	-	-	-	-	-	14	-0.64	-0.14
GO:0007267	Cell-cell signaling	-	-	-	-	-	37	-0.26	-0.32
GO:0042330	Taxis	-	-	-	-	-	17	-0.78*	-0.1
GO:0006935	Chemotaxis	-	-	-	-	-	17	-0.78*	-0.1
GO:0030574	Collagen catabolism	-	-	-	-	-	7	-0.69	-0.77

Significant differences are marked with an asterisk. DNA transp., DNA transposon; N, number of contributing genes; MCS, fractional intronic MCS density deviation (see text); IG, fractional igTEna deviation (see text).

**Table 3:** GO terms associated with TE-rich mouse genes

		Over-represented TE types											
		
GO term	Description	B1	L1	LTR	L2			MIR			B2/ID/B4		
	**Molecular function**				N	MCS	IG	N	MCS	IG	N	MCS	IG

GO:0005215	Transporter activity	-	-	-	64	-0.24	0.33	-	-	-	-	-	-
GO:0005216	Ion channel activity	-	-	-	-	-	-	28	0.2	-0.05	-	-	-
GO:0015268	Alpha-type channel activity	-	-	-	-	-	-	33	0.13	0.12	-	-	-
GO:0015267	Channel or pore class transporter activity	-	-	-	-	-	-	33	0.13	0.12	-	-	-
GO:0005261	Cation channel activity	-	-	-	-	-	-	23	0.37*	-0.08	-	-	-
GO:0005244	Voltage-gated ion channel activity	-	-	-	-	-	-	19	0.34	-0.1	-	-	-
	**Biological process**												
GO:0030001	Metal ion transport	-	-	-	-	-	-	26	0.31*	-0.06	-	-	-
GO:0007264	Small GTPase mediated signal transduction	-	-	-	-	-	-	-	-	-	14	-0.14	0.79

Significant differences are marked with an asterisk. N, number of contributing genes; MCS, fractional intronic MCS density deviation (see text); IG, fractional igTEna deviation (see text).
