# Supplementary material for: Gene function and expression level influence the insertion/fixation dynamics of distinct transposon families in mammalian introns
Source: Genome Biol. 2006 Dec 20;7(12):R120. doi: 10.1186/gb-2006-7-12-r120 (PMC1794433; doi:10.1186/gb-2006-7-12-r120)
Supplement: Additional data file 1 — Supplementary text presenting analysis of MCS density and TE integration frequency over evolutionary time. A supplementary figure describing the results is also provided (supplementary Figure 1) together with its legend. [file gb-2006-7-12-r120-S1.pdf]

Supplementary text

MCS density and TE integration: a matter of tempo.

It has been suggested [24, 33, 34] that TE insertion is constrained by the presence of multispecies conserved elements (MCS). Yet, TEs have been integrating over different time periods and MCS have also been fixed at various evolutionary ages. To gain further insight into the role of MCSs in affecting TE integration dynamics, we divided TEs in 10 percentile divergence (i.e. age) classes and calculated their frequency with respect to the inferred intron length at insertion time. The latter was estimated to be equal, at any age, to unique (TE-free) intron length plus intron size accounted for by older TEs. MCS fixation age was estimated as previously described [24] and conserved sequences were divided on the basis of their pre- or post-dating the bird-mammalian split. In particular, MCS that are conserved back to either *Fugu* or chicken were ascribed to the first group (old MCSs), while MCS that are conserved in human and mouse but not in birds or fishes were considered to belong to the second group (young MCSs). MCS-containing introns were then divided in old or young MCS density classes and TE insertion frequency at different ages was estimated [for partition of introns in MCS density classes quartiles in MCS density distribution (calculated on MCS-containing introns only) were used].

It is evident from Supplementary Figure 1A that old MCSs have been affecting TE integration over time, their effect being prevalent on older TEs. Conversely, young MCSs (Fig. 1B) have been playing small or no effects on the integration of old TEs since many of them probably became fixed either contemporarily or after insertion of older TE classes.

Supplementary Figure 1

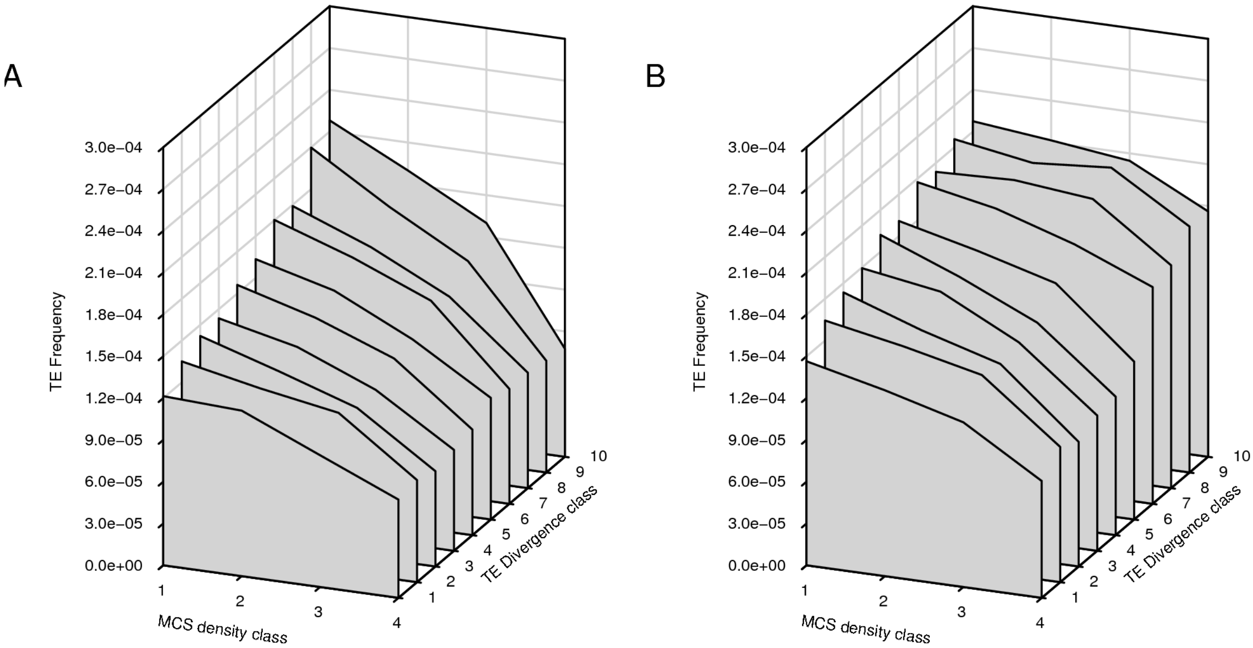

Analysis of TE integration over time. TE integration frequency was calculated, for each divergence class with respect to the inferred intron length at the time of insertion. MCS density classes were calculated (see methods) after dividing MCS on the basis of their pre- (A) or post (B) dating the bird-mammalian split.
