# Supplementary material for: Gene function and expression level influence the insertion/fixation dynamics of distinct transposon families in mammalian introns
Source: Genome Biol. 2006 Dec 20;7(12):R120. doi: 10.1186/gb-2006-7-12-r120 (PMC1794433; doi:10.1186/gb-2006-7-12-r120)
Supplement: Additional data file 2 — Supplementary Table 1 lists GO terms associated with mouse TE-poor genes. Supplementary Figure 2 shows analysis of murine MIR sequences associated with immune response genes. Supplementary Figure 3 shows gene-expression dependent variation in TE intronic abundance for human genes (SAGE data). Supplementary Figure 4 shows gene-expression dependent variation in TE intronic abundance for mouse genes (microarray data). Supplementary Figure 5 shows gene-expression dependent variation in TE intronic abundance for mouse genes (SAGE data). Supplementary Figure 6 shows intronic to intergenic relative frequency difference (calculated on gene flanks rather than entire intergenic regions). [file gb-2006-7-12-r120-S2.pdf]

Supplementary Table 1. GO terms Associated with mouse TE-poor genes

		UNDER-REPRESENTED TE TYPE																		
**GO term**	**Description**	**B1**			**B2/B4/ID**			**L1**	**L2**			**LTR**			**DNA trasposon**			**MIR**		
	**MOLECULAR FUNCTION**	N[a]	MCS[b]	IG[c]	N[a]	MCS[b]	IG[c]		N[a]	MCS[b]	IG[c]	N[a]	MCS[b]	IG[c]	N[a]	MCS[b]	IG[c]	N[a]	MCS[b]	IG[c]
GO:0005102	Receptor binding	112	0,43	-0,6 *	120	0,59	-0,42 *	-	-	-	-	163	0,32	0,28	159	0,26	-0,1	-	-	-
GO:0008083	Growth factor activity	46	1,05 *	-0,3	50	1,08 *	-0,38	-	-	-	-	55	0,86	0,46	-	-	-	-	-	-
GO:0005125	Cytokine activity	54	-0,01	-0,68	53	0,24	-0,42	-	-	-	-	76	0,01	0,55 *	-	-	-	-	-	-
GO:0005179	Hormone activity	-	-	-	32	0,34	-0,29	-	42	0,05 *	-0,01	36	0,22	-0,21	36	-0,04 *	-0,52	35	-0,07	0,04
GO:0003676	Nucleic acid binding	-	-	-	274	1,85 *	-0,57 *	-	-	-	-	383	1,09 *	-0,1	-	-	-	443	0,89	-0,46 *
GO:0003723	RNA binding	-	-	-	-	-	-	-	117	0,06	-0,45 *	-	-	-	-	-	-	95	-0,02	-0,19
GO:0030528	Transcription regulator activity	140	2,73 *	-0,41	153	2,5 *	-0,65 *	-	-	-	-	185	1,53 *	0,27	-	-	-	-	-	-
GO:0003700	Transcription factor activity	119	3 *	-0,51	128	2,8 *	-0,76 *	-	-	-	-	150	1,82 *	0,34 *	-	-	-	-	-	-
GO:0004871	Signal transducer activity	307	0,2	-0,59 *	299	0,33	-0,48 *	-	-	-	-	-	-	-	-	-	-	-	-	-
GO:0003735	Structural constituent of ribosome	-	-	-	-	-	-	-	116	-0,35 *	-0,55 *	91	-0,25 *	-0,54 *	100	-0,37 *	-0,71 *	107	-0,37 *	-0,85 *
GO:0005198	Structural molecule activity	-	-	-	-	-	-	-	-	-	-	157	-0,09	-0,28	172	-0,18 *	-0,5 *	168	-0,21 *	-0,49 *
	**BIOLOGICAL PROCESS**																			
GO:0050791	Regulation of physiological process	-	-	-	-	-	-	-	-	-	-	290	1,22 *	0,08	-	-	-	-	-	-
GO:0050789	Regulation of biological process	-	-	-	-	-	-	-	-	-	-	388	1,07 *	0,09	-	-	-	-	-	-
GO:0007275	Development	297	1,76 *	-0,23	287	1,89 *	-0,43 *	-	-	-	-	349	1,27 *	0,18	-	-	-	-	-	-
GO:0009653	Morphogenesis	179	1,74 *	-0,09	-	-	-	-	-	-	-	-	-	-	-	-	-	-	-	-
GO:0006952	Defense response	91	-0,16 *	-0,68 *	93	-0,11 *	-0,65 *	-	-	-	-	-	-	-	-	-	-	-	-	-
GO:0006955	Immune response	85	-0,12 *	-0,65 *	88	-0,07 *	-0,64 *	-	-	-	-	-	-	-	-	-	-	-	-	-
GO:0009607	Response to biotic stimulus	-	-	-	105	-0,11 *	-0,6 *	-	-	-	-	-	-	-	-	-	-	-	-	-
GO:0019222	Regulation of metabolism	-	-	-	-	-	-	-	-	-	-	281	1,26 *	0,08	-	-	-	-	-	-
GO:0006412	Protein biosynthesis	-	-	-	-	-	-	-	-	-	-	116	-0,11 *	-0,5 *	-	-	-	136	-0,24 *	-0,65 *
GO:0006139	Nucleobase, nucleoside, nucleotide and nucleic acid metabolism	-	-	-	-	-	-	-	-	-	-	-	-	-	-	-	-	435	0,75	-0,51 *
GO:0019219	Regulation of nucleobase, nucleoside, nucleotide and nucleic acid metabolism	-	-	-	-	-	-	-	-	-	-	263	1,32 *	0,08	-	-	-	-	-	-
GO:0006350	Transcription	-	-	-	-	-	-	-	-	-	-	273	1,25 *	0,04	-	-	-	-	-	-
GO:0006355	Regulation of transcription, DNA-dependent	-	-	-	-	-	-	-	-	-	-	252	1,34 *	0,09	-	-	-	-	-	-
GO:0006351	Transcription, DNA-dependent	-	-	-	-	-	-	-	-	-	-	256	1,32 *	0,07	-	-	-	-	-	-
GO:0045449	Regulation of transcription	-	-	-	-	-	-	-	-	-	-	261	1,33 *	0,09	-	-	-	-	-	-
GO:0006820	Anion transport	44	-0,22	-0,75 *	-	-	-	-	-	-	-	-	-	-	-	-	-	-	-	-
GO:0015698	Inorganic anion transport	43	-0,21	-0,71 *	-	-	-	-	-	-	-	-	-	-	-	-	-	-	-	-

Supplementary Figure 2

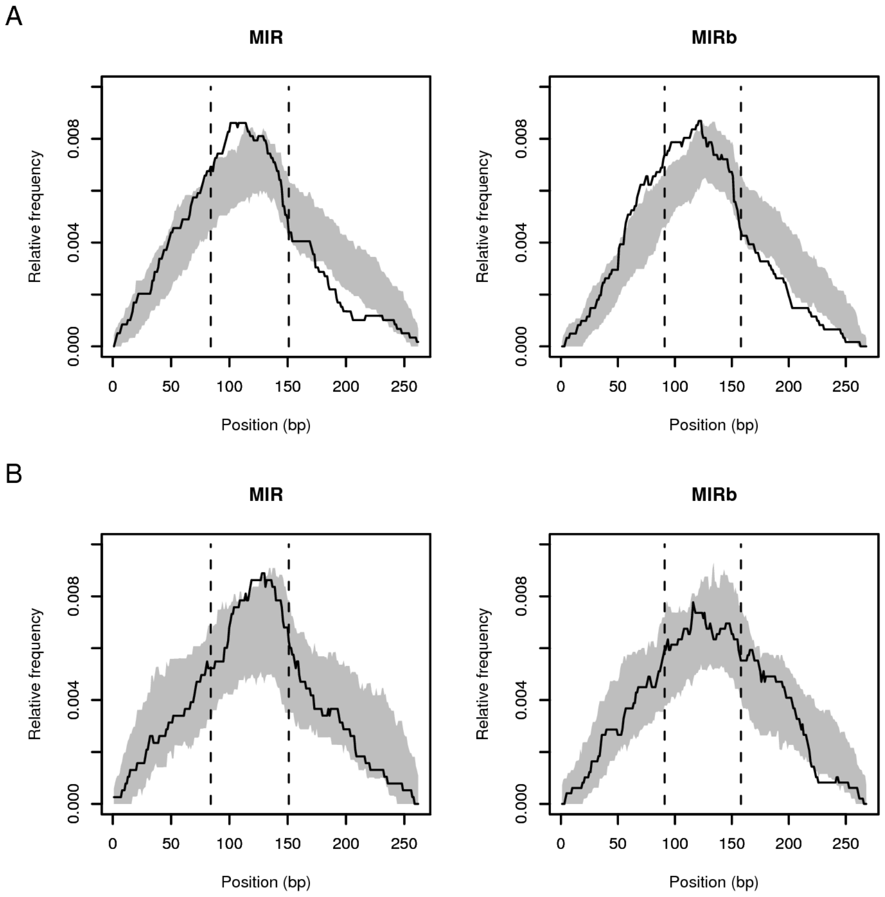

Analysis of murine MIR sequences associated with immune response genes. (A) Relative frequency at each position of MIR (n= 59) and MIRb (n= 63) consensus sequences in immune response gene introns (black lines). Frequency intervals corresponding to the 1st and 99th percentiles in 100 random sample frequency distributions are represented by the grey areas. (B) the same as in (A) for MIRs located in intergenic regions. MIR, n= 46; MIRb, n= 63. The same calculations were not performed for MIR3 and MIRm sequences because of their paucity.

Supplementary Figure 3

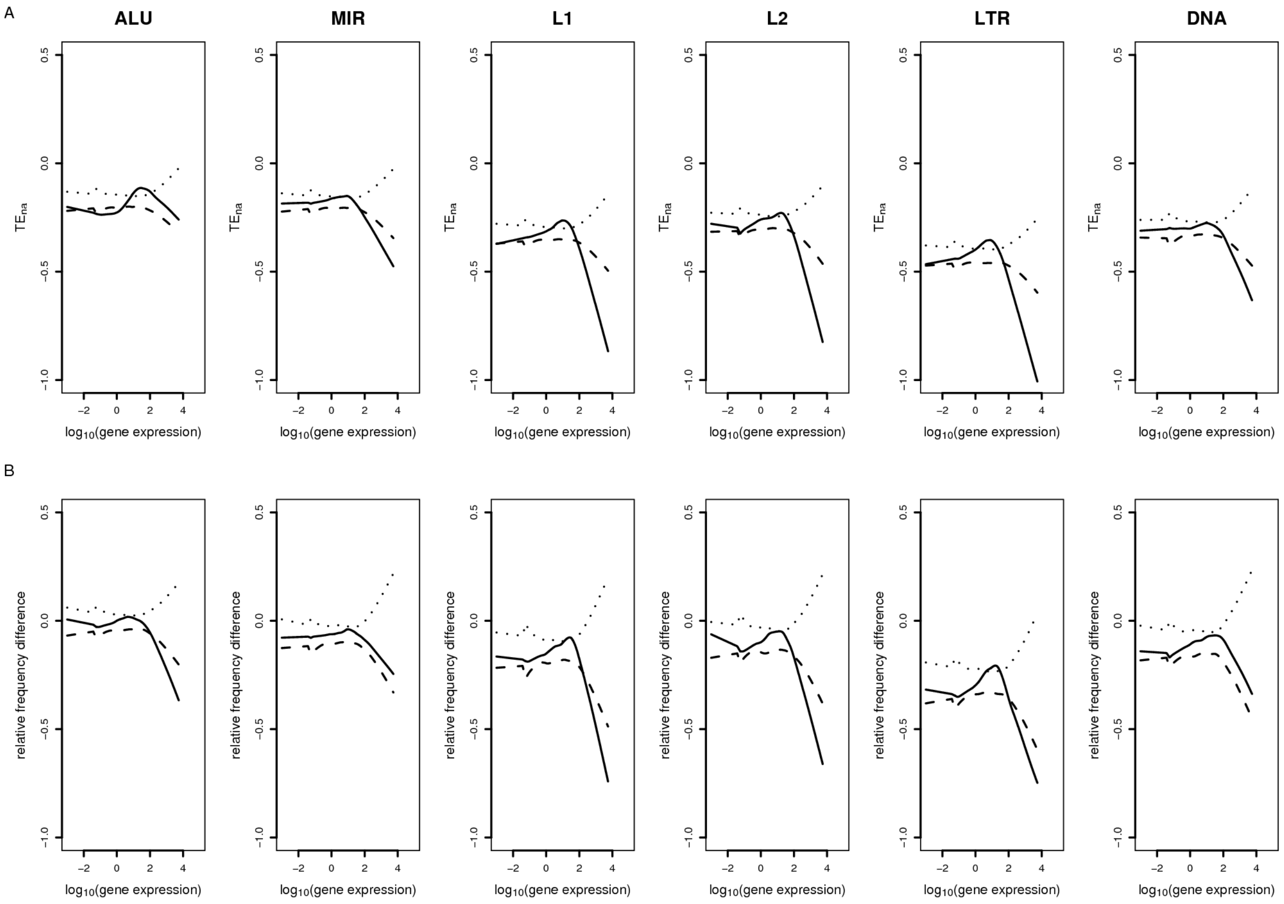

Gene-expression dependent variation in TE intronic abundance for human genes. Gene expression levels were derived from SAGE data. (A) Lowess fit (solid line) and probability intervals (hatched lines) of TEna versus gene expression level (log transformed values) for the six TE families. (B) Lowess fit (solid line) and probability intervals (hatched lines) of intronic to intergenic relative TE frequency difference (see text) versus gene expression level (log transformed values).

Supplementary Figure 4

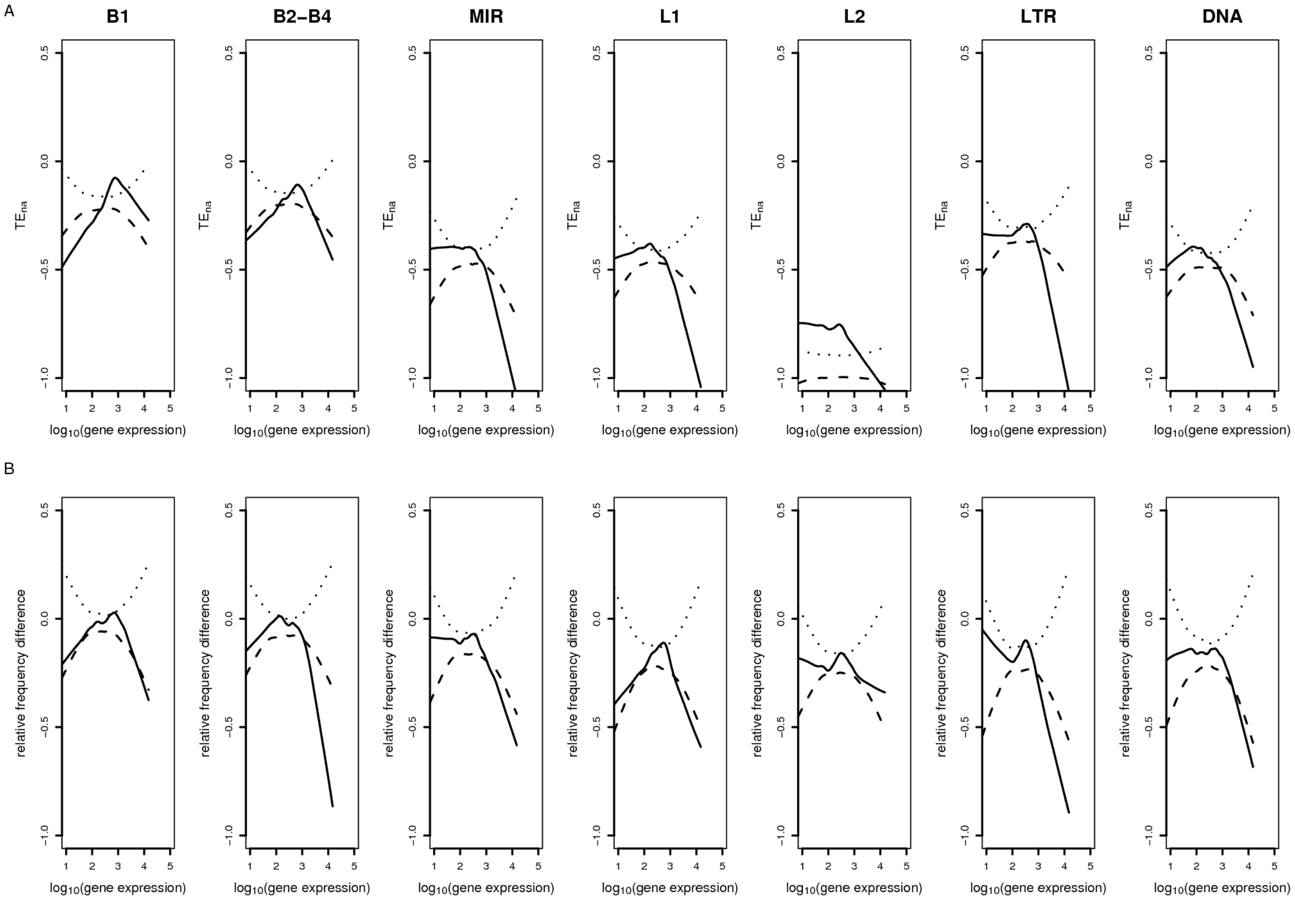

Gene-expression dependent variation in TE intronic abundance for mouse genes. Gene expression levels were derived from microarray data. (A) Lowess fit (solid line) and probability intervals (hatched lines) of TEna versus gene expression level (log transformed values) for the six TE families. (B) Lowess fit (solid line) and probability intervals (hatched lines) of intronic to intergenic relative TE frequency difference (see text) versus gene expression level (log transformed values).

Supplementary Figure 5

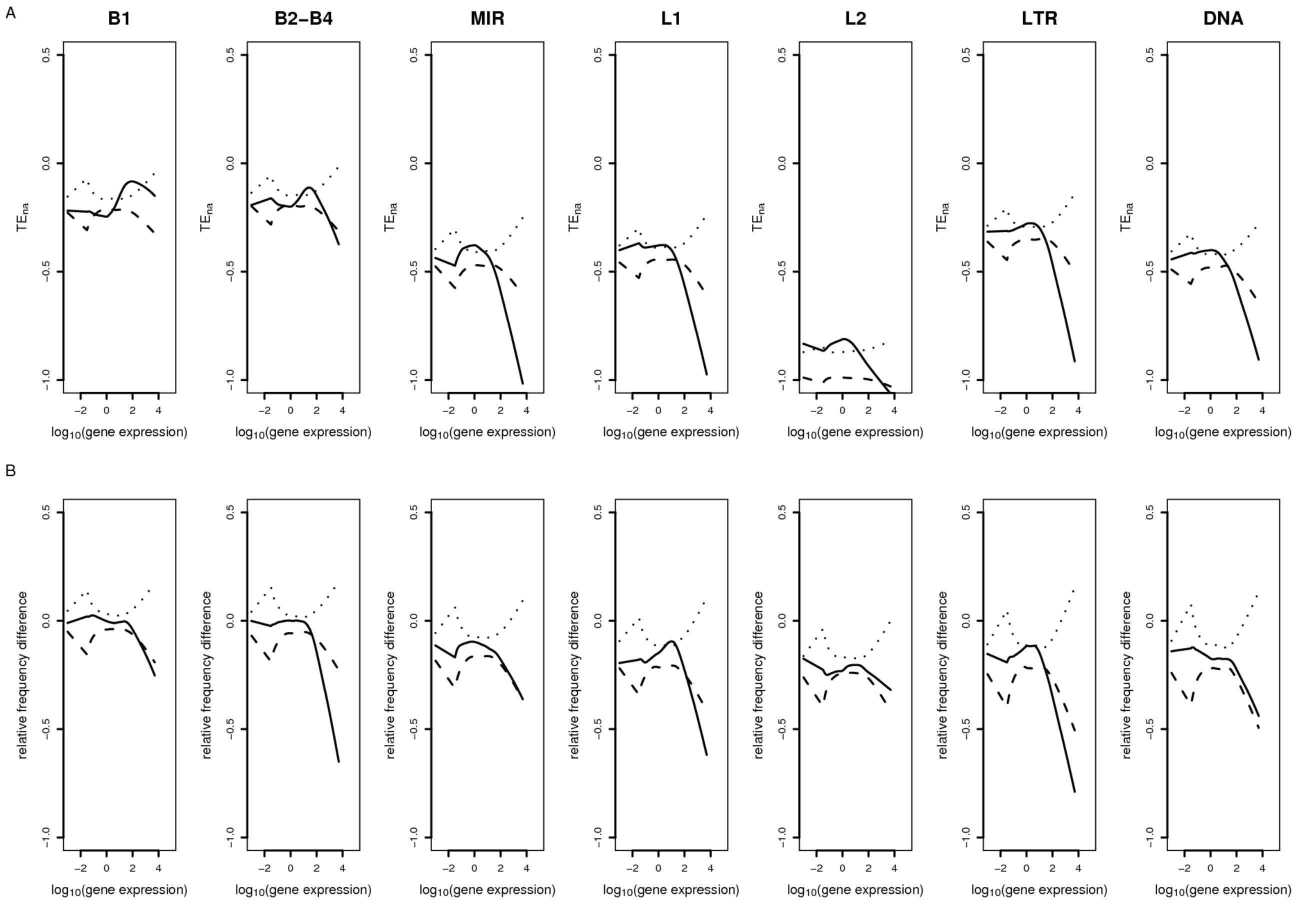

The same as for supplementary figure 4 except expression data were derived from SAGE experiments.

Supplementary Figure 6

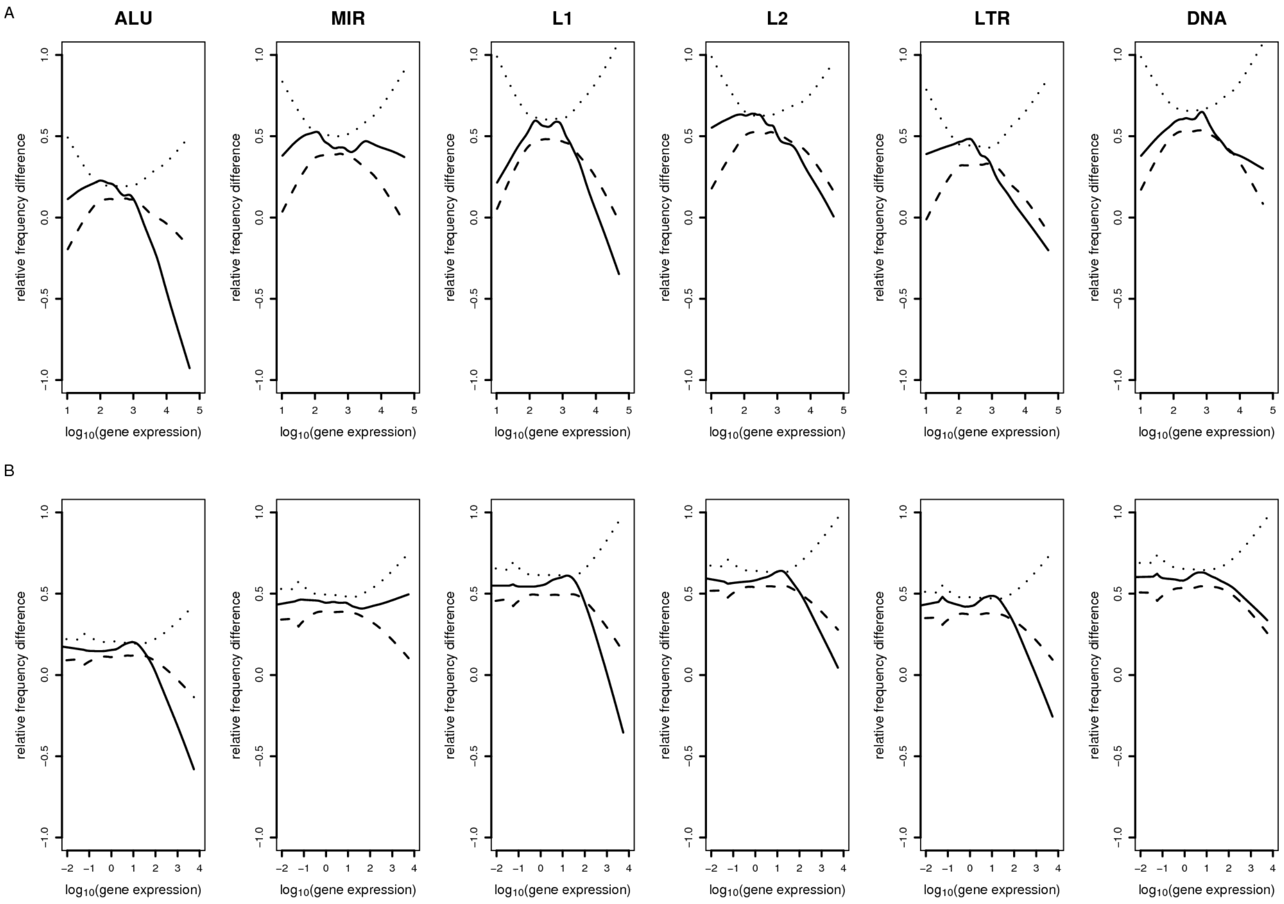

Lowess fit (solid line) and probability intervals (hatched lines) of intronic to intergenic relative TE frequency difference (see text) versus gene expression level (A, microarray data; B, SAGE data). In this case, TE frequency was calculated in the 4 kb flanking both gene sides (8 kb total) instead of in entire intergenic regions.
